# Supplementary material for: In-Situ Metatranscriptomic Analyses Reveal the Metabolic Flexibility of the Thermophilic Anoxygenic Photosynthetic Bacterium Chloroflexus aggregans in a Hot Spring Cyanobacteria-Dominated Microbial Mat
Source: Microorganisms. 2021 Mar 21;9(3):652. doi: 10.3390/microorganisms9030652 (PMC8004040; doi:10.3390/microorganisms9030652)
Supplement: Supplementary file 1 [file microorganisms-09-00652-s001.zip › Supplemental_materials/Supplemental_figures.docx]

*In-situ* metatranscriptomic analyses reveal the metabolic flexibility of the thermophilic anoxygenic photosynthetic bacterium *Chloroflexus* *aggregans* in a hot spring cyanobacteria-dominated microbial mat

Shigeru Kawai^1,2^*, Joval N. Martinez^1,3^, Mads Lichtenberg^4^, Erik Trampe^4^, Michael Kühl^4^, Marcus Tank^1,5^, Shin Haruta^1^, Arisa Nishihara^1,6^, Satoshi Hanada^1^, and Vera Thiel^1,5^*

^1^ Department of Biological Sciences, Tokyo Metropolitan University, Hachioji, Tokyo 192–0397 Japan

^2^ Institute for Extra-cutting-edge Science and Technology Avant-garde Research (X-star), Japan Agency for Marine-Earth Science and Technology (JAMSTEC), Yokosuka, Kanagawa 237-0061 Japan

^3^ Department of Natural Sciences, College of Arts and Sciences, University of St. La Salle, Bacolod City, 6100 Negros Occidental, Philippines

^4^ Department of Biology, Marine Biological Section, University of Copenhagen, Strandpromenaden 5, DK 3000 Helsingør, Denmark

^5^ DSMZ – German Culture Collection of Microorganisms and Cell Culture, GmbH Inhoffenstraße 7B 38124 Braunschweig, Germany

^6^ Bioproduction Research Institute, National Institute of Advanced Industrial Science and Technology (AIST), Ibaraki 305-8566, Japan

***** Correspondence: [kawais@jamstec.go.jp](mailto:kawais@jamstec.go.jp), Vera.Thiel@DSMZ.de

**Figure S1.** Relative transcription levels of paralogous respiratory complex I. Mean values of relative transcripts of two sets of genes encoding respiratory complex I (1 and 2) are shown with standard deviation. The complex I-1 (Cagg_1620-1631, *blue line*) is composed of 12-genes comprising cluster with an additional *nuoM* (2-M complex) inserted between the original *nuoM*1 and nuoN genes (*nuoABCDHJKLMMN*) and the complex I-2 (Cagg_1036-1049, *orange line*) represents a complete gene set containing 14 genes (*nuoABCDEFGHIJKLMN*). The downwelling photon irradiance (PAR; 400-700 nm) is indicated in *white*.

**Figure S2.** Relative transcription levels of housekeeping genes. Mean values for relative transcriptional levels of genes encoding DNA gyrase (Cagg_2331 and 3685), RNA polymerases (Cagg_0050, 0201, 0898, 0905, 0979, 1357, 1599, 2227, 2614, 2706-2707, 2716, 2997, 3145 and 3845) and DNA polymerases (Cagg_0019, 0978, 2156, 2499, 2589, 2973, 3199, 3590 and 3714) are represented by a *blue line*, an *orange line*, and *yellow line*, respectively, with standard deviations. The downwelling photon irradiance (PAR; 400-700 nm) is indicated in *white*.

**Figure S3.** Relative transcription levels of genes related to oxidative stresses. The values of relative expression levels are respectively displayed for glutathione peroxidase 1 and 2 (Cagg_0324, *blue line*; 0446, *orange line*) and superoxide dismutase (Cagg_2494, *yellow line*). Mean value of the glycolate oxidase *glcDEF* (Cagg_1528, 1530-1531, and 1892-1893, *green line*) is shown with standard deviations. The downwelling photon irradiance (PAR; 400-700 nm) is indicated in *white*. The asterisk indicates the transcription of a particular gene corresponding to the color in a timepoint differed significantly from that in the previous timepoint.

**Figure S4.** Relative transcription levels of amino acid and oligopeptide transporter genes. Mean values of relative transcripts of transporter genes encoding general L-amino acid transporter (*aapPMQJ*: Cagg_0335-0338, *blue line*), branched-chain amino acid transporter (single *livK*, Cagg_2427 and two paralogs of *livHMGF*, Cagg_2425-2526, 2423 and 2428, and Cagg_1862-1859, *orange line*), and two paralogs of oligopeptide transporter (*oopABCDF*: Cagg_2385-2383 and Cagg_2965-2964, *yellow line*) are shown with standard deviations. The downwelling photon irradiance (PAR; 400-700 nm) is indicated in *white*.

**Figure S5.** Relative transcription levels of glycoside and sugar transporter genes. Mean values of relative transcripts of glycoside and sugar transporter genes encoding of arabinogalactan oligomer/maltooligosaccharide transporter (homologous *ganOPQ*-I and II, Cagg_0406-0404 and Cagg_3183-3181, *blue line*), alpha-glucoside transporter (*aglEFG*: Cagg_3716-3718, *orange line*), putative multiple sugar transporter (*chvE* and *gguAB*: Cagg_2576-2578, *yellow line*), and general nucleoside transporter (*bmpA* and homologous *nupCBA*-I and II: Cagg_3761, Cagg_0429-0431, Cagg_3847 and Cagg_3175-3176, *green line*) are shown with standard deviations. The downwelling photon irradiance (PAR; 400-700 nm) is indicated in *white*.
